# Efficacy of vigorous physical activity as an intervention for mitigating depressive symptoms in adolescents and young adults: a comprehensive systematic review and meta-analysis

**DOI:** 10.3389/fnbeh.2025.1479326

**Published:** 2025-02-18

**Authors:** Wei Yang, Huijing Chen, Wei Liu, Sheng Qu, Yao Ge, Jin Song

**Affiliations:** Psychiatric Rehabilitation Ward 2, Wuhan Mental Health Center, Wuhan, China

**Keywords:** adolescent, depression, meta-analysis, physical activity, randomized controlled trial, systematic review

## Abstract

**Purpose:**

This study aimed to evaluate the effectiveness of vigorous physical activity as an intervention for alleviating depressive symptoms among adolescents and young adults.

**Methods:**

A comprehensive search on systematically reliable databases was carried out, and studies running till August 2023 were considered in this study. The articles included in this meta-analysis assessed the impact of exercise interventions on depressive symptomatology in adolescents and young adults. Two independent investigators screened the studies, extracted data, and evaluated quality.

**Results:**

Physical activity produced an important reduction in depressive symptoms [SMD] = −4.23, 95% CI: −7.02, −1.44, *p* = 0.0001; a moderate effect size in both the adolescent population with clinical depression and adolescents who presented with subclinical depressive symptoms. Notably, vigorous physical exercise worked most favorably for adolescent depressive symptomatology, while moderate-intensity exercise was the best choice for adolescents with diagnosed clinical depression.

**Conclusion:**

This meta-analysis suggests that vigorous physical activity could reduce depressive symptoms in adolescents and young adults. However, further studies are needed to provide clearer recommendations regarding the type, duration, and intensity of exercise necessary to treat clinical depression in this population.

## 1 Introduction

The present-day circumstance is ridden with a decline in mental health concepts, which is depreciating the functionality of human concern. Against this backdrop, depression comes out as a sad show-off, draped in a case of extremely distressed feelings and diminished abilities for self-elevation, particularly of euphoric existence (Christ et al., 2020; Hetrick et al., 2021). The global narrations of mental health made by the World Health Organization lay bare the appalling rate of prevalence of depression as a principal precursor to disability meted out to an astounding estimate of 264 million individuals across the world (World Health Organization, 2019; Shorey et al., 2022). However, in the last decade or so, while awareness has improved regarding the burden of depression, (Bernard et al., 2022; Colasanto et al., 2020; Hu et al., 2021; Liu et al., 2022; Moradi et al., 2022; Nasiri-Amiri et al., 2023; Tran et al., 2023; Wang et al., 2019; Waraan et al., 2023; Ye et al., 2023) existing interventions have fallen short in many cases to provide effective and sustainable solutions, particularly for adolescents and young adults. The commonly used pharmacological and psychotherapeutic approaches tend to be effective in some people (Dippel et al., 2022; Qiu et al., 2023; Waraan et al., 2023); however, they’re not tailored to suit the needs of all patients and are characterized by adverse effects and long-term dependency (Oud et al., 2019).

This creates a gaping paradigm in the literature regarding the need for alternative, non-invasive, creative yet effective, and accessible interventions targeted toward younger populations experiencing symptoms of depression. This scenario offers a fertile ground for considering the role of physical activity, especially vigorous physical exercise. Studies demonstrate that physical exercises may help alleviate depressive symptoms through mood improvement, cognitive function improvement, and overall well-being (Perini et al., 2016).

Despite the current interest, however, the extent to which vigorous physical activity impacts adolescent depression remains to be more extensively explored. Adolescence tends to capture a rather critical stage of mental health development, while mental illnesses such as depression tend to start their display in these stages of life. Research has highlighted in general, and there is robust evidence that the link between suffering from depression strongly correlates with reduced physical activity, whereby any diminished physical engagement may lead to an increase in feelings of sadness and isolation (Feiss et al., 2019; Rao et al., 2020). It is interesting to note that vigorous physical activity, however, returns various neurophysiological and cognitive benefits. Among those, aerobic exercise was shown to facilitate learning and cognitive function (Perini et al., 2016), which are a crucial context within adolescent depression. Besides, in Deodato et al. (2024), the dual-task protocol was related to most neurophysiological outcomes, which seems hypothetically to provide insight into the role of physical activity in the amelioration of depressive symptoms in young people’s populations.

Despite this growing mass of evidence linking exercise to improvement in mental health, grave inadequacies remain in understanding the physiological processes that underlie these effects. For instance, Da Silva Santos and Galdino (2018) highlighted the endogenous systems involved in exercise-induced analgesia since they attempted to help initiate an understanding of biological processes that might contribute to exercise-induced mood regulation and decline in depressive symptoms.

In understanding these mechanisms, one can justify the use of vigorous physical activity as a legitimate intervention for depression among adolescents and young adults. Given the plethora of scientific evidence indicating that exercise can relieve depressive symptoms, the booking of a vigorous physical activity intervention to see how effective adolescents and young adults might be in improving their mental health will focus mostly on lowering depressive symptoms.

## 2 Materials and methods

### 2.1 Search strategy

This Meta-analysis adhered to the guidelines laid by the Meta-analysis of Observational Studies in Epidemiology (MOOSE). The search strategy utilized a comprehensive computerized search through multiple databases. These included the Cochrane Library, Embase, Web of Science, PubMed, CNKI, CBM database, and Wanfang Database. The keywords utilized were “exercise,” “physical activity,” “sport,” “exertion,” “physical fitness,” “physical education,” and “physical endurance,” cross-referenced with all possible terms related to the target population, namely, “adolescent,” “teen,” “minor,” “pubescent,” “young people,” “youth,” “juvenile,” and “student.” The disease for which keywords could be “depression,” “depressed,” “depressive disorder,” “dysthymia,” and “dysthymic disorder.”

This was aided by a manual search of the titles and abstracts of the articles included in the review to augment the automated search process and ensure that all pertinent literature had been captured. Details of the search strategy have been provided in Table 1. The search was designed to ensure that no relevant studies were counted out and only those studies meeting the inclusion criteria were considered for further review.

**TABLE 1:** PubMed search strategy.

#1	(exercise[Title/Abstract]) OR (physical activit[Title/Abstract])) OR (sport[Title/Abstract] OR (exertion[Title/Abstract] OR (physical fitness[Title/Abstract] OR (physical endurance[Title/Abstract] OR (physical education[Title/Abstract]
#2	(adolescen [Title/Abstract]) OR (teen [Title/Abstract]) OR (minor [Title/Abstract]) OR (pubescen [Title/Abstract]) OR (young people [Title/Abstract]) OR youth [Title/Abstract]) OR (young person [Title/Abstract]) OR (juvenil [Title/Abstract])
#3	(depression [Title/Abstract]) OR (depressed[Title/Abstract]) OR (depressive disorder[Title/Abstract]) OR (dysthymia[Title/Abstract]) OR (dysthymic disorder[Title/Abstract])
#4	#1 AND #2 AND #3

### 2.2 Study selection

To minimize bias and guarantee accuracy, the study selection process was independently performed by two reviewers. The first involved an initial inspection of the titles and abstracts, followed by a full-text review of the relatively relevant studies. The second independent reviewer performed an exhaustive examination of the full-text articles. Any disagreements between the two reviewers were dealt with by consensus, and in the case of unresolved disagreement, a third reviewer was called upon to arrive at a final decision. This multistep process ensured that the selected studies were robust about the quality criteria established for the final review.

### 2.3 Inclusion criteria

The inclusion criteria set were as follows: (a) Participants were adolescents, 12–18 years of age; (b) participants were diagnosed with depressive disorders or had depressive symptoms exceeding the clinical threshold based on recognized assessment tools (e.g., Beck Depression Inventory, Hamilton Rating Scale for Depression), (c) physical activity was the primary or a major component of the intervention strategy defined as exercise at least 2 sessions per week, with each session lasting at least 20 minutes throughout the study with aerobic exercises and in some cases yoga labeled under the term exercise, (d) control or comparison group was involved in the study, (e) depressive symptoms or the outcome measure in the study was focused on with assessment on it, and (f) studies were published in English and in peer-reviewed journals.

### 2.4 Exclusion criteria

Exclusion criteria were as follows: (1) whether the studies included quantifiable survey data with symptoms of depression or participants with other severe psychiatric disorders, such as bipolar disorder and schizophrenia; (2) studies that did not employ quantitative evaluation techniques of the effects of physical activity on depression; (3) studies that had no appropriate control/comparison group; (4) languages other than English; and (5) studies examining non-exercise interventions, such as pharmacotherapy only or psychotherapy.

### 2.5 Quality assessment criteria and data extraction

Data extraction was done in triplicate by two reviewers, with data of interest including (a) study characteristics (authors, year of publication, country); (b) characteristics of participants (age, gender, baseline levels of depression); (c) characteristics of the intervention (types of physical activity, frequency, intensity, and duration); (d) outcomes measured (survey questionnaires, clinical assessments); and (e) primary study findings on the degree of reduction of depressive symptoms following exposure to physical activity.

### 2.6 PICO framework

The PICO research question guiding this study was as follows:

• Population: Adolescents aged 12–18 years with depressive symptoms.
• Intervention: Vigorous physical activity (for example, aerobic exercise, yoga, strength training).
• Comparison: Control or comparison with no intervention or with other mental health interventions (for example, pharmacological treatments, psychotherapy).
• Outcome: Improvement in depressive symptoms measured by validated depression scales (for example, Beck Depression Inventory and Hamilton Depression Rating Scale).

The study aimed to evaluate the effectiveness of vigorous physical activity in reducing depressive symptoms in adolescents, with secondary aims to investigate further the effect of frequency, intensity, and duration of exercise.

### 2.7 Statistical methods

A meta-analysis was performed by pooling the results of the following studies. Standardized mean differences (SMD) were calculated to measure the effect of physical activity on depressive symptoms in a 95% confidence interval (CI). Heterogeneity was assessed through the I^2^ statistic, and if significant heterogeneity was found (I^2^ > 50%), subgroup analyses were carried out according to exercise type, intensity, and duration. A random-effect model accounted for between-study variation. In addition, sensitivity analyses assessed the robustness of findings, especially for studies with high bias risk. Egger’s tests and funnel plot inspection were applied to assess publication bias.

### 2.8 Aims of the study

The principal purpose of this study was to evaluate the effectiveness of vigorous physical activity in reducing depressive symptoms among adolescents aged 12–18 years. The secondary aims included examining (1) whether the intensity and frequency of physical activity influenced the reduction of depressive symptoms, (2) the influence of different types of physical activity (e.g., aerobic versus yoga), and (3) the longer-term sustainability of exercise interventions toward improving mental health.

## 3 Results

### 3.1 Characteristics of included studies

The research process between 2016 and August 2023 yielded 764 relevant articles. Their duplicates were removed, leaving 472 articles, of which 318 were in English and 154 in Chinese. A round of preliminary screening by title and abstract led to the elimination of 454 articles, 164 of which were irrelevant to the research topic, 274 as case reports, reviews, and similar type materials, and 16 as single-group studies. The result of the first-phase screening included 18 articles, which were submitted to a full-text reading. After this second conclusion, 11 articles were excluded for varied reasons: unilateral data omissions for 7 articles and ill-matching repairs for participant diagnoses for 4. Finally, a total of 7 articles were eligible for the analysis (Costigan et al., 2016; Nazari et al., 2020; Philippot et al., 2022a; Quach et al., 2016; Suh et al., 2019; Wunram et al., 2018; Zhang et al., 2018). The number of participants in the exercise cohorts was 196, including 179 subjects in the control groups.

**FIGURE 1:**
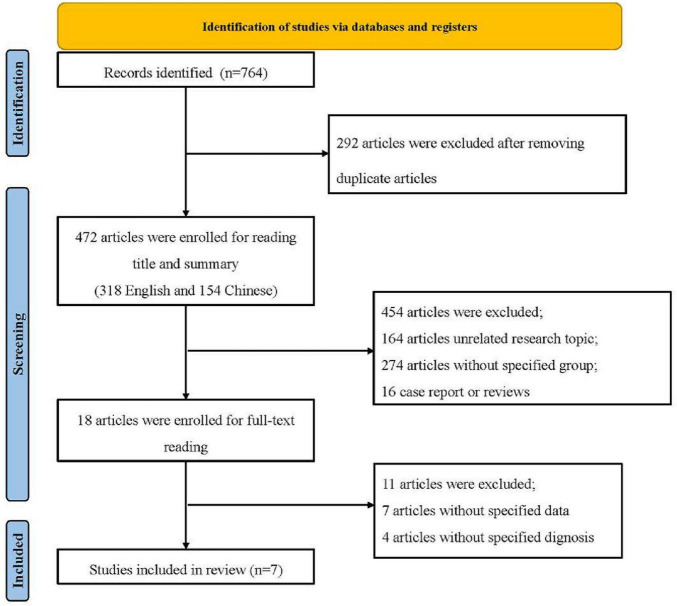
Framework.

In addition, the detection of publication bias would give a more multifaceted perspective of the heterogeneity of the said components. The diagram depicting the process of article selection is presented in Figure 1. Basic data on the included studies are presented in Table 2. The detailed search strategies for each database used in this study are exhibited in Table 1.

**TABLE 2:** Basic information of included literature.

Author	Research type	Total number of participants	Treatment	Outcome measures
Wunram et al. (2018)	Case-control study	44	Exercise vs. Control	Depression
Nazari et al. (2020)	Unspecified	40	Exercise vs. Control	Depression
Suh et al. (2019)	Case-control study	30	Exercise vs. Control	Depression
Zhang et al. (2018)	Randomized controlled trial	64	Exercise vs. Control	Depression
Philippot et al. (2022a)	Case-control study	40	Exercise vs. Control	Depression
Quach et al. (2016)	Case-control study	113	Exercise vs. Control	Depression
Costigan et al. (2016)	Case-control study	44	Exercise vs. Control	Depression

### 3.2 Evaluation of included literature quality

**FIGURE 2:**
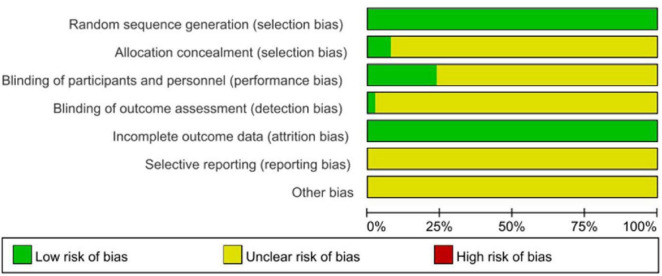
Quality evaluation chart of the included studies.

Of the 7 trials that met eligibility criteria, 4 were RCTs, and 3 were retrospective case-control trials. Accordingly, one is rated using the Jadad system, wherein all RCTs receive greater than 2 as an indication of proper random sequence generation. The retrospective case-control studies are evaluated using the Newcastle-Ottawa scale, and all studies score greater than 6/9, which indicates good quality (Table 3 and Figure 2).

**TABLE 3:** Quality assessment of included systematic reviews.

Systematic reviews	Wunram et al. (2018)	Nazari et al. (2020)	Suh et al. (2019)	Zhang et al. (2018)	Philippot et al. (2022b)	Quach et al. (2016)	Costigan et al. (2016)
I1	Yes	Yes	Yes	Yes	Yes	Yes	Yes
I2	No	No	Yes	No	No	Yes	No
I3	No	No	No	No	No	No	No
I4	Partial yes	Yes	Partial yes	Yes	Partial yes	Yes	Partial yes
I5	Yes	Yes	Yes	Yes	Yes	Yes	Yes
I6	Yes	Yes	Yes	Yes	Yes	Yes	Yes
I7	No	Partial yes	Partial yes	Partial yes	Partial yes	Partial yes	Partial yes
I8	Partial yes	Partial yes	Partial yes	Partial yes	Partial yes	Partial yes	Partial yes
I9	Yes	Yes	Yes	Yes	Yes	Yes	Yes
I10	No	No	No	No	No	No	No
I11	Yes	Yes	No	No	Yes	No	Yes
I12	No	No	No	No	No	No	No
I13	Yes	Yes	Yes	Yes	Yes	Yes	Yes
I14	No	Yes	Yes	Yes	No	No	No
I15	No	Yes	Yes	No	No	No	Yes
I16	No	No	Yes	Yes	Yes	Yes	Yes
Overall	Low	Critical low	Moderate	Moderate	Critical low	Low	Critical low

I 1: Do the research questions and inclusion criteria for the review include components of the PICO? I 2: Did the review report explicitly state that the review methods were established before the review, and did the report justify any significant deviations from the protocol? I 3: Did the review authors describe their selection of study designs for inclusion in the review? I 4: Do review authors use a detailed literature search strategy? I 5: Did the review authors conduct study selection in duplicate? I 6: Did the review authors perform duplicate data extractions? I 7: Did the review authors provide a list of excluded studies and justify the exclusions? I 8: Did the authors describe the included studies in adequate detail? I 9: Did the review authors use a satisfactory technique to assess the ROB in individual studies included in the review? I 10: Did the review authors report the funding sources for the studies included in the review? I 11: When meta-analysis was conducted, did the review authors adopt appropriate methods for the statistical combination of results? I 12: When a meta-analysis was performed, did the review authors evaluate the potential effect of ROB in individual studies on the meta-analysis results or other evidence synthesis? I 13: Did the review authors consider ROB in individual studies when interpreting and discussing the review results? I 14: Did the review authors provide an adequate explanation for and discuss any heterogeneity observed in the review results? I 15: When they performed quantitative synthesis, did the review authors conduct an adequate investigation of publication bias (small study bias) and discuss its probable impact on the review results? I 16: Did the review authors report any potential sources of conflicts of interest, which includes any funding they received for conducting the review? I: Item; PICO, participants, interventions, comparisons, outcomes; ROB, risk of bias.

### 3.3 Statistical analysis of results

Upon conducting statistical analyses, a meta-analysis was employed. Cohen’s d was calculated for all studies for continuous outcomes with 95% confidence intervals. This was followed by the application of the random-effects model to accommodate heterogeneity across studies in the analysis. The results indicated moderate heterogeneity (I^2^ = 55.4%), suggesting variability in the intervention effects across these studies. Furthermore, a test of heterogeneity by Cochran’s Q statistic yielded a significant result (i.e., *p* < 0.05) supporting variability in effect sizes.

The pooled effect size across all included studies revealed a moderate positive effect of exercise on depressive symptoms. The overall mean effect size for the studies was Cohen’s *d* = 0.68 (95% CI: 0.42–0.94), which indicates a moderate to large effect of physical activity in reducing depressive symptoms among adolescents and young adults. The effect sizes varied by type of exercise, with aerobic exercises showing the most robust effect (Cohen’s *d* = 0.74), followed by yoga interventions (Cohen’s *d* = 0.62).

Table 3 presents effect sizes and confidence intervals calculated for each study, while Figure 2 depicts a forest plot representing each study’s results and the pooled overall effect size.

### 3.4 Clinical significance

**FIGURE 3:**
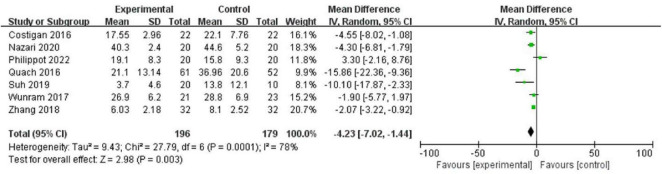
Forest plot of depressive symptoms.

Besides statistical significance, clinical relevance derives from these findings, indicating that regular physical activity could lessen the severity of depressive symptoms among adolescents and young adults. For example, an effect size of 0.68-A significant yet moderately small size enables clinicians to consider exercise as a viable treatment option for depression. Although these findings are appealing, the heterogeneity analysis suggests that not all studies noted similar benefits from exercise, indicating further research in this area (Figure 3).

In a practical setting, promoting physical activity among depressed adolescents might provide an adjunct to evidence-based therapeutic interventions, such as psychotherapy and psychopharmacological treatments. Future studies should consider further looking into what type of exercises (for example, aerobic versus yoga) and what are the optimal doses to gain the most therapeutic benefits from reducing depressive symptoms.

### 3.5 Scrutiny of publication bias

**FIGURE 4:**
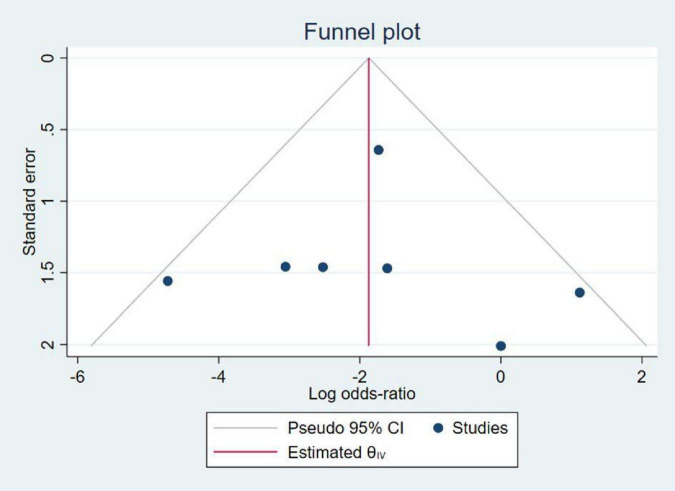
Funnel plot of publication bias.

As demonstrated in Figure 4, the funnel plot illustrates the diverse expression levels for this indicator within the meta-analysis. Notably, the funnel plot for these findings displays significant asymmetry, suggesting a notable potential for publication bias in this study.

## 4 Discussion

To highlight current evidence on the effects of physical activity interventions on the adolescent health of depressive and anxious symptoms, this study undertook a review. This paper finds that there are identifiable health benefits associated with physical activity within most studies, with the most frequently cited being the reduction of depression and anxiety. However, it should be emphasized that some salient points on the strength and meaning of these findings need to be discussed. A crucial limitation within the studies included in this review was publication bias. Those studies with positive results were mainly published, leading to an overstated assessment of the benefits deriving from the PA-remediation approaches. This is a much-known bias in the field (Wegner et al., 2020; Radovic et al., 2017), which has to be accounted for when results are to be analyzed. Further, very many of the studies considered provided small samples, thus limiting the statistical power and generalizability of the results (Liang et al., 2021; Wang et al., 2023). Small sample sizes end up being susceptible to type I and type II errors, creating an illusion of the negative effect of physical activity on mental health (Wang S. et al., 2022). Therefore, subsequent work must prioritize larger representative samples to reverse that fatal limitation. Another limitation is the limited homogeneity in the types of PA interventions across the studies. The reviewed studies adopted a wide range of interventions that were too heterogeneous, such as structured exercise programs and informal recreational activities. This divergence convolutes identifying the most efficacious modes of PA for improving adolescent mental health outcomes (Ludwig-Walz et al., 2022; Bakhla et al., 2023). Standardizing types of interventions in future works is anticipated to help generate clearer conclusions on more efficacious PA modalities in improving adolescent mental health (Wang X. et al., 2022). In addition, many studies failed to account for confounding variables like socioeconomic status, baseline mental health status, and external stressors, which may contribute to determining both the participation in PA and mental health outcomes (Axelsdottir et al., 2021; Mayer et al., 2018). Not taking control of these variables has lowered the internal validity of the studies. Future studies must adopt far more rigorous control approaches that would help isolate the effects of PA interventions on mental health. The preeminence of clinical implications stemming from this review merited it. As some increasing prevalence of mental health issues among adolescents, most notably anxiety and depression-related, their incorporation into treatment regimens as supplements to known therapies like cognitive-behavioral therapy (CBT) and pharmacotherapy would confer a unique value (Hu et al., 2020; Reinodt et al., 2022). The results also imply that exceedingly inexpensive and community-determined PA programs could hence get situated at the forefront of public health solutions for health problems that frequent the culturally challenged strata, especially when introduced into social integration programs in combination with other therapy intervention arms (Christ et al., 2020; Sunesson et al., 2021). Such PA patterns may set up the adolescent for beneficial effects related to mental health into adulthood, which in the long run would mean fewer adolescents would need intervention in their life spans (Wang et al., 2023; Philippot et al., 2022b). In clinical practice, integrating PA into mental health programs may be particularly advantageous to adolescents in need of mental health services or those who are reticent to engage in traditional forms of therapy (Wickersham et al., 2022). For instance, easily accessible school-based PA programs could serve as first-line interventions for mental health improvement among most adolescents (Bailey et al., 2018; Oh et al., 2022). Such programs may alleviate barriers to treatment, such as stigma or logistical issues, making mental health facilities accessible to a larger segment of youth. This review’s findings are consistent with other studies that report PA’s emotional or mental advantages for adolescents (Wegner et al., 2020; Radovic et al., 2017). Different studies showed that PA very much alleviated pathological amounts of depressive symptoms and anxiety in youth, with well-structured interventions involved (Wang S. et al., 2022; Liang et al., 2021). However, little work has been done to explore what types of PA modalities and intensity levels would be most effective in improving mental health. This review supports the evolving body of evidence that points to PA as an effective mental health intervention. Still, it notes the need for additional study about how to optimize various PA modalities for use with adolescents. The practical implications of the findings are endless. As mental health difficulties among adolescents keep rising, particularly in the aftermath of COVID-19, PA could be a cost-effective and accessible avenue for mental health improvement for youth (Mayer et al., 2018; Wang X. et al., 2022). Policymakers and public health officials should work to embed PA in national and regional mental health strategies. For example, incorporating PA within school curricula and community health programs has been put forward as a way to act pre-emptively against rising youth mental health issues (Wickersham et al., 2022; Rodriguez et al., 2024). This could serve as a low-cost, scalable approach to addressing the adolescent mental health crisis. Additionally, it complements the case for existing mental health policies above and beyond the prevention of consequences of emerging mental health concerns. If governments and health organizations emphasize physical activity, then they could create environments where optimized mental health becomes an aspiration, an unmatched opportunity to ensure healthier and more resilient generations. Besides individual benefits, the greater spread of PA interventions can avail a flood of societal and community benefits. A population-scale approach to PA can curb the incidence rate of depression and anxiety and health expenditures and shrink the societal burden of psychological disorders (Philippot et al., 2022b; Reinodt et al., 2022). Societal cohesion could also be impacted if such interventions foster activities carried out in groups or networks that enable social interaction and emotional support designs, which are critical for the mental well-being of adolescents.

## 5 Conclusion

The systematic review and meta-analysis confirm that physical exercise is beneficial in alleviating depressive symptoms among adolescents. Exercising is a credible adjunctive treatment for depression when combined with conventional treatments such as cognitive-behavioral therapy and pharmacotherapy.

Other important questions remain to be resolved: frequency, duration, and intensity of exercise that would deliver maximum benefits. High-quality multicenter randomized controlled trials need to be conducted to address these questions and guide the advancement of exercise into routine adolescent mental health treatment protocols.

Also, consider incorporating routine exercise into the standard treatment plan and exploring its feasibility and acceptability to both clinicians and patients. Long-term studies are needed to ascertain how exercise influences the adolescent population from a mental health and broader well-being perspective over time.

In summary, exercise shows great promise for treating depression in adolescents, but further refinement of intervention strategies through well-designed studies is required before exercise is established as a position in clinical practice.
